# Robust Multiple-Range Coherent Quantum State Transfer

**DOI:** 10.1038/srep28886

**Published:** 2016-07-01

**Authors:** Bing Chen, Yan-Dong Peng, Yong Li, Xiao-Feng Qian

**Affiliations:** 1 College of Electronics, Communication & Physics, Shandong University of Science and Technology, Qingdao 266510, China; 2 Beijing Computational Science Research Center, Beijing 100094, China; 3 Center for Coherence and Quantum Optics, and The Institute of Optics, University of Rochester, Rochester, NY 14627, USA

## Abstract

We propose a multiple-range quantum communication channel to realize coherent two-way quantum state transport with high fidelity. In our scheme, an information carrier (a qubit) and its remote partner are both adiabatically coupled to the same data bus, i.e., an *N*-site tight-binding chain that has a single defect at the center. At the weak interaction regime, our system is effectively equivalent to a three level system of which a coherent superposition of the two carrier states constitutes a dark state. The adiabatic coupling allows a well controllable information exchange timing via the dark state between the two carriers. Numerical results show that our scheme is robust and efficient under practically inevitable perturbative defects of the data bus as well as environmental dephasing noise.

Quantum state transfer (QST) in many-body solid state physical systems plays a central role in the realization of various localized quantum computation and quantum communication proposals^1^,^2^,^3^. A practical high quality quantum state transfer scheme needs to possess several desirable features: i) high fidelity (to preserve the transferred message), ii) robustness (to tolerate inevitable practical errors, imperfections, and decoherence), iii) efficiency (to achieve optimal results with minimal implementations), and iv) flexibility (to serve for multiple tasks). The investigation of accomplishing high fidelity quantum state transfer in electronic and spin systems has recently drawn tremendous attention (see for example refs 4, 5, 6, 7, 8, 9, 10, 11, 12, 13, 14, 15, 16, 17 and an overview^3^). Many of these schemes are based on the natural dynamical evolution of permanent coupled chain of quantum systems, and require no control during the QST. However, such schemes rely on precise manufacture of the system interaction parameters as well as accurate timing of information processing, and may not be robust against experimental imperfection settings, such as small variations of the system Hamiltonian, environmental noise, etc.

Recently, adiabatic passage has been paid much attention for quantum information transfer in various physical systems^18^,^19^,^20^,^21^,^22^,^23^,^24^,^25^,^26^,^27^,^28^,^29^,^30^,^31^,^32^,^33^,^34^,^35^,^36^,^37^,^38^,^39^,^40^,^41^,^42^,^43^,^44^,^45^,^46^. One typical way is called coherent QST which involves a quantum system that has an instantaneous eigenstate that is a superposition of a message state and its corresponding target state. Stimulated Raman adiabatic passage (STIRAP)^18^ is such an example in a three-level atomic system. In this technique, the dark state which is a coherent superposition of message and target states plays a central role in the process of information transfer. In STIRAP evolution, the message and target states are coupled to the same intermediate state by a pump pulse and a Stokes pulse respectively. If the two pulses are applied counter-intuitively, i.e., the Stokes pulse is applied before the pump, then the dark state is associated initially with the message state and eventually with the target state. Such a process effectively transports the information of the message state to the desired target state. There are a couple of advantages of the adiabatic passage scheme: it is robust against small errors and imperfections of settings, and the QST timing can be controlled freely and precisely. However, in atomic systems, the message is only transferred from one state to another of the same local atom, and is incapable to realize non-local long-range information transport.

In this paper we extend the STIRAP protocol for the first time to an *N*-site tight-binding model and show that it is suitable for high fidelity robust QST and quantum information swapping from one location (quantum dot site) to another [see schematic illustration in Fig. 1(a)]. The tight-binding quantum dot (QD) array with a single diagonal defect serves as the adiabatic pathway for two-way electronic transport. Two external QDs A, B represent information sender and receiver or vice versa are allowed to be flexibly side coupled to the array (i.e., QDs A B can couple to different sites of the QD array as required by particular tasks). In this scheme, the coupling parameters between the external QDs and the corresponding sites on the array are made time dependent, which are controlled by the sender and receiver, respectively. We find that the ground state of the array is a bound state (or localized state) due to the existence of the defect. This allows us to show analytically that our system is an effective three-level system when the coupling between QD A (B) and the QD array are weak. As a consequence it is demonstrated that high fidelity two-way QST can be realized at various different distances between the sender and receiver. Numerical results are also performed to illustrate that our scheme is robust against dephasing and small variations of the QD couplings and imperfections.

## Results

### Driven Model

**Figure 1:**
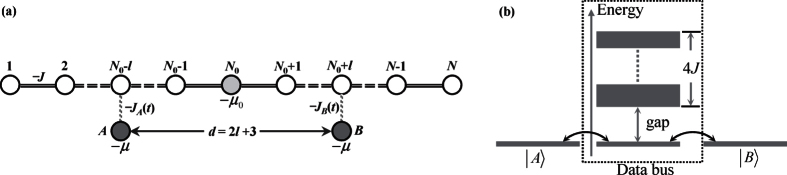
Schematic illustrations of multiple-range adiabatic quantum state transfer from A to B. (**a**) The tight-binding array with single defect is acting as a quantum data bus, in which the coupling strengthes −*J* are time-independent and the defect QD is supplied with energy −*μ*_0_. The sender (QD *A*) and the receiver (QD *B*) supplied with on-site energy, −*μ* are coupled to two sites of the array on opposite sides with respect to the defect site. The sender controls −*J*_*A*_(*t*) and the receiver controls −*J*_*B*_(*t*). The transfer distance in terms of the number of sites is given as *d* = 2*l* + 3. (**b**) Schematic illustration of the energy spectrum of the data bus. The defect contributes one out-of-band energy and form a non-vanishing gap between two lowest energy level. By ensuring that two outmost QDs are resonantly coupled to the zeroth energy mode of the data bus, quantum state transfer becomes analogous to three-level scheme.

**Figure 2:**
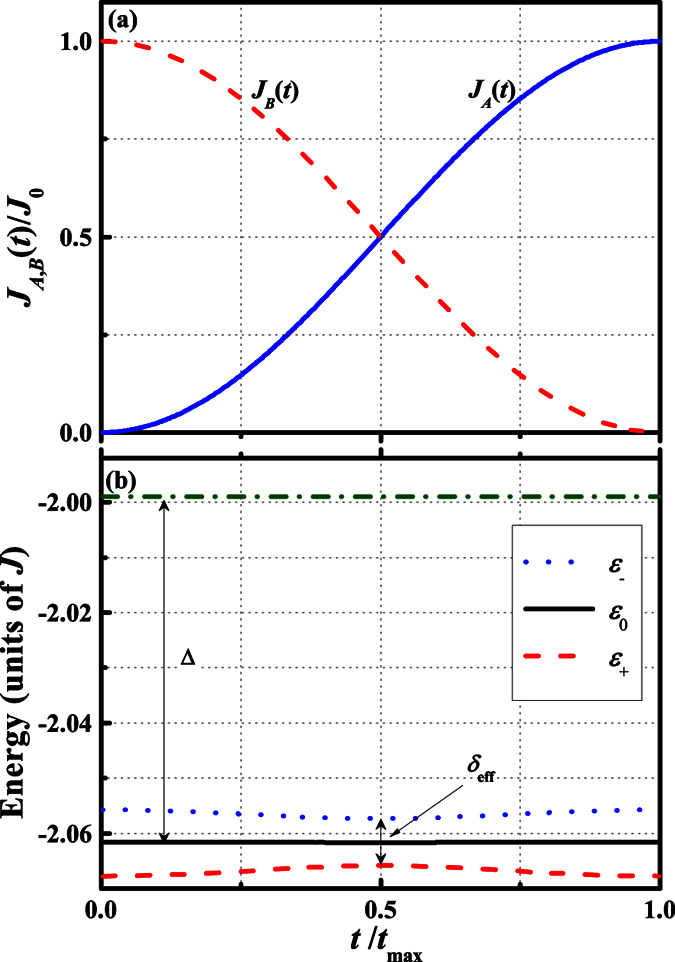
Plots of system parameters and time-dependent eigenenergies. (**a**) The time-dependent tunnelling rates *J*_*A*_(*t*) and *J*_*B*_(*t*) as a function of time (in units of *J*_0_), *J*_*A*_(*t*) is the solid line and *J*_*B*_(*t*) is the dashed line. The pulse sequence is applied in the counter-intuitive order. (**b**) The instantaneous eigenenergy (in units of *J*) of the lowest four eigenstates of total Hamiltonian (1) through the pulse shown in (**a**), which were obtained by direct numerical diagonalization of the Hamiltonian. In the weak coupling limit, i.e. *J*_0_ ≪ *J* three lowest states is approximately equivalent to that a triple-quantum-dot system.

We start with our proposed scheme illustrated in Fig. 1(a): the channel connecting the two side QDs *A* and *B* is a one-dimensional tight-binding array with uniform and always-on exchange interactions and with one diagonal defect at *N*_0_th site. The coupling between the QDs A, B and their corresponding channel sites are made time dependent. The total Hamiltonian can be written in the following structure:


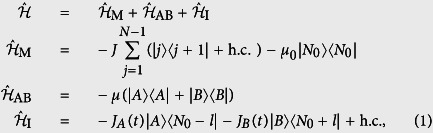


where −*J* (<0) is the coupling strength between nearest neighboring QDs along the channel; *J*_*A*_(*t*) is the time dependent coupling strength between *A* and the (*N*_0_ − *l*)th site of the QD array and is controlled by the sender, while *J*_*B*_(*t*) is the receiver controlled coupling strength between *B* and site (*N*_0_ + *l*); −*μ*_0_ and *μ* are the on-site energy applied on the QDs; 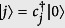 represents the Wannier state localized in the *j*-th quantum site for *j* = *A*, 1, 2, ..., *N*, *B*. For convenience, we consider the channel containing an odd number of QDs and the gate voltage −*μ*_0_ is applied on the central dot, i.e. *N*_0_ = (*N* + 1)/2. Note that equation (1) comprises three terms: the first corresponds to the tight-binding chain with defect at *N*_0_-site, the second is the energy of QDs A, B, and the third term describes the interaction Hamiltonian. In this paper we study the electron transfer from QD-*A* to QD-*B* through the tight-binding array serving as quantum data bus, and we denote the transfer distance *d* = 2*l* + 3. In this proposal the propagation of the electron is driven by two time-dependent coupling strengths, i.e., *J*_*A*_(*t*) and *J*_*B*_(*t*), which are modulated in sinusoidal pulses


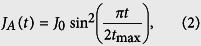



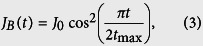


where *t*_max_ is the prescribed duration of QST; *J*_0_ is the maximum tunnelling rates between the two external QD and the defected chain. These two pulses are illustrated in Fig. 2(a).

As shown in Method section, the *δ*-type diagonal defect in Hamiltonian 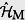 contributes only one bound state 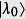 with energy 
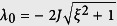
. As schematically shown in Fig. 1(b), there exists a non-vanishing energy gap between two lowest eigenstates


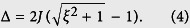


Two external QDs A, B represent information sender and receiver or vice versa, which are allowed to be flexibly side coupled to the tight-binding array as the aim required. We here restrict our discussion to tuning the on-site energy of QDs A, B to match exactly that of the localized state 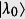, i.e., 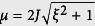. Thus, the two end spins are resonantly coupled to the localized state mode by 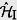. For weak coupling case, the total system can be mapped to an effective three-level system


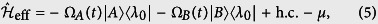


where Ω_*α*_(*t*) is the effective coupling strength between state 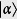 and mode 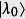 for *α* = *A*, *B*. The eigenstates of the Hamiltonian of the Eq. (5) are


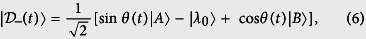



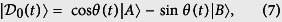



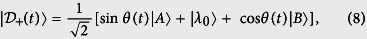


where *θ*(*t*) = arctan[Ω_*A*_(*t*)/Ω_*B*_(*t*)]. The effective Hamiltonian and corresponding eigenstates are derived in detail in Methods section. From the fact that the system is driven by an adiabatic process, the coupling strengths *J*_*A*_(*t*) and *J*_*B*_(*t*) are slowly varying due to the relative flatness (controlled via large *t*_max_) of the pulse shape. Therefore the eigenstates above are of the same form as the time-independent ones. The validity of these states are confirmed by our exact numerical results that will be shown later.

In Fig. 2(b), we plot the four lowest eigenenergies of total Hamiltonian (1) using the pulsing scheme given by Eqs (2) and (3) in weak coupling regime. To first order in *J*_0_, the perturbation Hamiltonian 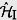 lifts degeneracy of the ground-state manifolds while the excited states 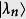 are unaffected. This observation is schematically shown in Fig. 2(b), in which Δ is the typical gap for the unperturbed Hamiltonian 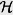 (i.e., *J*_0_ = 0) and the energy splitting *δ*_eff_ of the ground-state subspace is proportional to *J*_0_. In fact, the weak coupling limit yields the inequality *δ*_eff_ ≪ Δ which will be equivalent condition for the purposes of perturbation.

Bearing in mind the effective Hamiltonian (5) is the analytic approximation of the total Hamiltonian (1) and this approximation holds when the energy splitting *δ*_eff_ caused by the 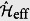 is smaller than the typical gap for the unperturbed Hamiltonian 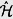. We now investigate the range of validity about the above approximation in the following, and *J*_0_ and *μ*_0_ are scaled by *J* for simplicity.

We compare the instantaneous eigenstate 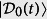 at time *t* = *t*_max_/2 of 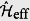 with the density matrices reduced from the first excited states of the total system. The density matrix corresponding to 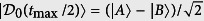 is


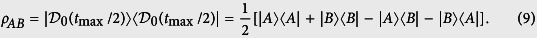


**Figure 3:**
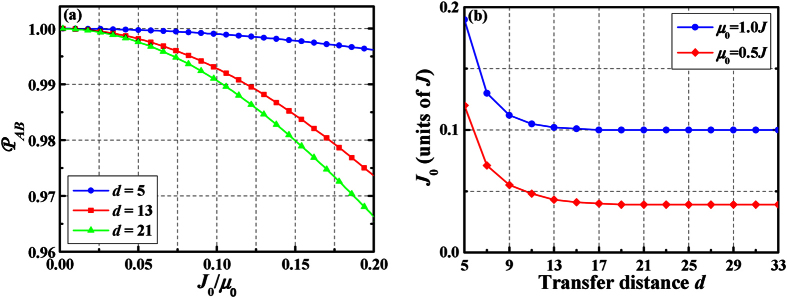
(**a**) The operator fidelity 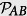 as a function of *J*_0_/*μ*_0_ for *d* = 5, 13, and 21. As the ratio *J*_0_/*μ*_0_ increases the operator fidelity is decreased. (**b**) The coupling *J*_0_ as a function of *d* for *μ*_0_ = 0.5*J*, and 1.0*J* (bottom to top along *J*_0_ axis) under the condition that the operator fidelity greater than 99.5%. The other system parameter is *N* = 39.

Moreover, we assign the state 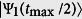 denotes the instantaneous first-excited state for the total Hamiltonian 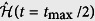. Then, the operator fidelity is defined as


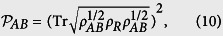


where 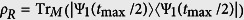, and Tr_*M*_ means the trace over the variables of the tight-binding array. 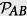 is sensitive to two parameters, i.e., the ratio of *J*_0_/*μ*_0_ and the transfer distance *d*. In the following discussions, we will investigate how the above external parameters influence the operator fidelity of the dark state. Figure 3(a) shows the dependence of 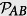 on both *J*_0_/*μ*_0_ and *d* for the system with *N* = 39. We can see that the operator fidelity improves by decreasing *J*_0_/*μ*_0_. Moreover, we note that, increasing the value of *d* there is a slight shift of 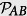 for a given *J*_0_/*μ*_0_, which means that as the transfer distance increases one need to decrease the ratio *J*_0_/*μ*_0_ if we want high operator fidelity to hold. To obtain high quality of operator fidelity (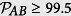
%), we compute the ratio *J*_0_/*μ*_0_ versus transfer distance *d* with two different impurity on-site energy *μ*_0_; these results are shown in Fig. 3(b). In this figure, one can see that taking *J*_0_/*μ*_0_ ≤ 0.1, the effective Hamiltonian agrees very well with the exact solution obtained from numerical calculations. Therefore, we have shown the weak coupling effective Hamiltonian (5) is a very good approximation of the exact model.

### Adiabatic transport

In this section, we investigate the process of information transport between QDs *A* and *B* with adiabatic passage. We begin with our investigation from one of the three eigenstates of the effective three-level Hamiltonian


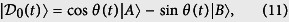


which can serve as the vehicle for population transfer in a STIRAP-like procedure. As described in Methods, the angle *θ*(*t*) is totally dependent on the ratio of the two pulse strength and the pulse sequence used here are in the counterintuitive ordering. It is well known that, for the counterintuitive sequence of pulses, in which *J*_*B*_(*t*) precedes *J*_*A*_(*t*), one has *θ* = 0 for *t* = 0 and *θ* = *π*/2 for *t* = *t*_max_. With these results, one can see that the state 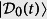 is 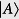 initially and goes to 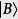 finally. The goal here is to study the coherent quantum state transfer from state 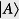 to state 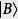 by slowly varying the alternating pulse sequence between QDs A, B and the chain to drive the state transfer. Here we define the transfer distance *d* to be the number of QDs between the two QDs which the sender A and receiver B are respectively connected with, i.e., *d* = 2*l* + 3. The transfer fidelity depends on two aspects: (i) the validity of the effective Hamiltonian (5) which is derived from perturbative way and (ii) the dynamics follows the instantaneous eigenstate 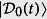 is whether or not adiabatic.

To realize high-fidelity QST, we require that the system remains in its dark state 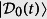, without loss of population from this state to the neighboring states, i.e., 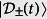. The adiabaticity parameter defined for this scheme is


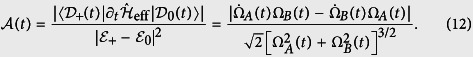


**Figure 4:**
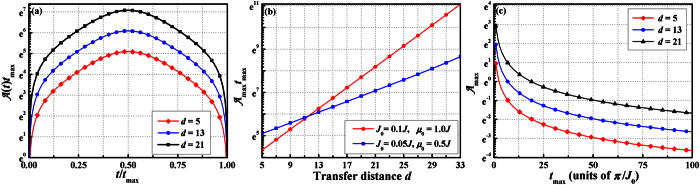
Plots of adiabaticity as a function of three factors. (**a**) Adiabaticity 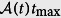 as a function of time in the time interval *t* ∈ [0, *t*_max_] corresponding to the pulse shapes as in Eq. (2). The results show that the adiabaticity parameter 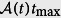 is largest at the crossing point of the two pulses. The parameters we chosen are *N* = 39, *J*_0_ = 0.05*J*, and *μ*_0_ *=* 0.5*J*. (**b**) Maximum adiabaticity 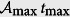 through the protocol as a function of transfer distance *d* for *N* = 39. The parameters is *J*_0_ = 0.05*J*, *μ*_0_ = 0.5*J* (squares) and *J*_0_ = 0.1*J*, *μ*_0_ = 1.0*J* (circles). As the transfer distance increases, the adiabaticity parameter increases and the grow of adiabaticity of the large *μ*_0_ is faster than the small one. (**c**) The maximum adiabaticity parameter 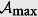 as a function of *t*_max_. As *t*_max_ is increased 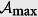 is decreased, indicating that better fidelity transfer can be achieved for longer total transfer time.

The time dependence of the parameter 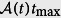 is illustrated in Fig. 4(a). Obviously the adiabaticity parameter 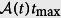 reaches maximum at the crossing point of the two pulses. At this point one has Ω_*A*_(*t*_max_/2) = Ω_*B*_(*t*_max_/2) = *J*_0_*u*_0_(*N*_0_ − *l*)/2 and 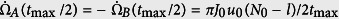. By using the form of the pulses as given in Eq. (2), the above equation gives rise to the simple form


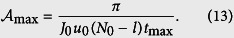


The analytical expression for maximum adiabaticity is helpful for estimating the quantum state transfer time *t*_max_. For adiabatic evolution of the system we require 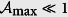. According to Eq. (13), the total transfer time should satisfy *t*_max_ ≫ *π*/[*J*_0_*u*_0_(*N*_0_ − *l*)]. In Fig. 4(b) we present the dependence of the 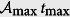 on the transfer distance *d* for a system with *N* = 39. Obviously one sees the increase in the adiabaticity parameter with increasing *d*. Moreover, one can see that the bigger is the *μ*_0_, the faster is the growth of 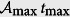. The reason is that by increasing *μ*_0_, the localization effect of Eq. (28) is enhanced. Figure 4(b) also reflects the fact that the optimal transfer time needs to be increased with increasing *d*. The smaller the *μ*_0_ is, the slower the growth rate will be. Furthermore, the dependence of the adiabaticity parameter on the total protocol time *t*_max_ is plotted in Fig. 4(c). With longer *t*_max_, and hence lower 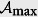, the transported electron is more likely to remain in the desired 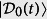 state resulting in better fidelity transfer.

To simulate the analog of STIRAP protocol we initialize the device so that the particle occupies site 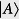 at *t* = 0, i.e., the total initial state is 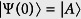, and apply the alternating pulse sequence [see Eq. (2)] in the counterintuitive order. The evolution of the wave function is described by Schrödinger equation


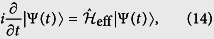


which creates a coherent superposition: 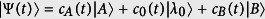. Substituting the superposition form of 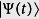 into the Schrödinger equation, we get equations of motion for the probability amplitudes


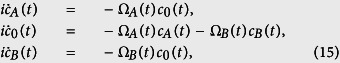


where the dot denotes the time derivative.

A measure of the quality of this protocol after the pulse sequences is given by the transfer fidelity


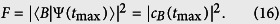


**Figure 5:**
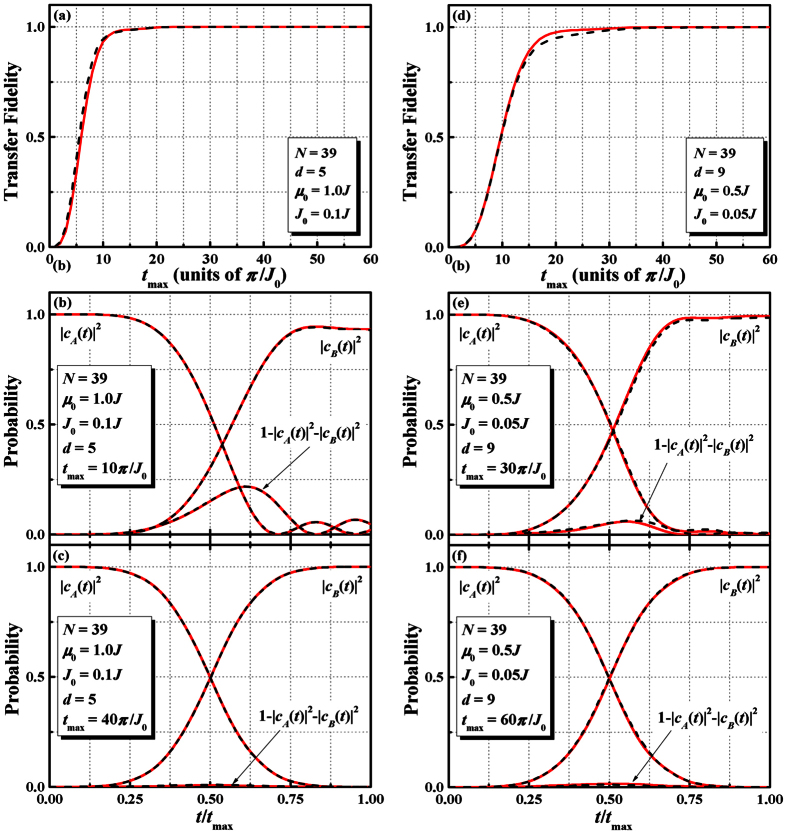
Plots of state transfer via adiabatic passage. (**a**) The transfer fidelity *F* as a function of *t*_max_ (in units of *π*/*J*_0_) for *d* = 5. The parameters we take is *N* = 39, *μ*_0_* *= 1.0*J*, and *J*_0_ = 0.1*J*. The red solid curves correspond to the approximate results via perturbation theory and black dotted curves are the exact numerical results for the complete Hilbert space. (**b**) The time evolution of the probabilities induced by the pulses in Fig. 2(a) for *d* = 5 and *t*_max_ = 10*π*/*J*_0_. (**c**) is the same as (**b**) but for *t*_max_ = 40*π*/*J*_0_. (**d**) The same as in (**a**), but for *d* = 9, *μ*_0_ = 0.5*J,* and *J*_0_ = 0.05*J*. (**e**–**f**) is sam**e** as (**b,c**) but for *d* = 9. To get high fidelity transfer, more time is required for longer transfer distance.

To investigate QST between QDs *A* and *B*, we numerically solve the time-dependent Schrödinger equation for the multi-dot system with *N* = 39. Here we compare the results obtained from two alternative approaches. In the first case, we numerically integrated Eq. (14) with the initial condition 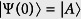. In the second case, we adopt total Hamiltonian (1) to replace the effective Hamiltonian and we perform a numerical simulation using the total Hamiltonian. In this case the computation takes place in full Hilbert space with basis 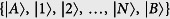. Figure 5(a) shows transfer fidelity *F* as a function of *t*_max_ for *d* = 5, *μ*_0_ = 1.0*J* and *J*_0_ = 0.1*J*. If we choose *t*_max_ ≥ 19*π*/*J*_0_, the transfer fidelity *F* will be larger than 99.5%. To illustrate the process of QST, we exhibit in Fig. 5(b,c) the time evolution of the probabilities. We get perfect state transfer if we choose the transfer time longer enough and the populations on the QD *A* and QD *B* are exchanged in the expected adiabatic manner. For the case *d* = 9 with *μ*_0_ = 0.5*J* and *J*_0_ = 0.05*J*, it is shown in Fig. 5(d–f) that to ensure *F* ≥ 99.5% the optimal transfer time is about 31*π*/*J*_0_. For comparison, we also plot in Fig. 5 the result of the exact numerical results (dashed curves) for the full Hilbert space calculation. Obviously, our three-state effective Hamiltonian describes the quantum state evolution very well.

**Figure 6:**
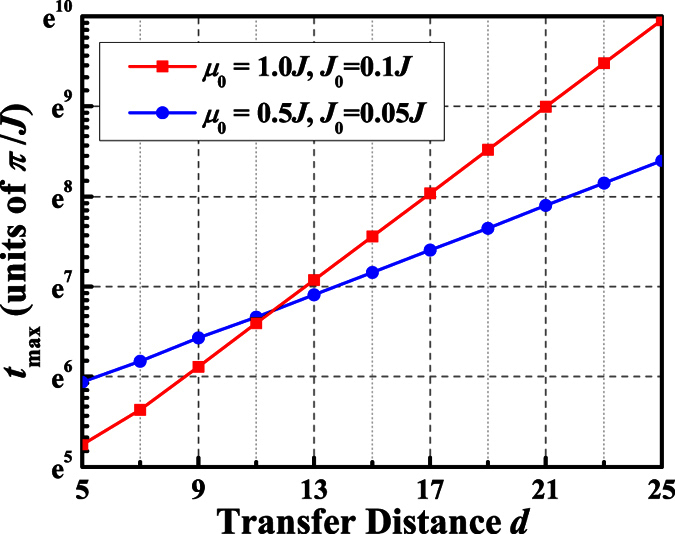
The plot of distance dependence of transfer time *t*_max_ for a chosen tolerable transfer error 1 − *F* = 0.5%. The lines are only guides to the eyes. Notice the exponential increase of *t*_max_ as a function of the distance. In order to make results comparable, all times are scaled in units of *π*/*J*.

In order to provide the most economical choice of total transfer time *t*_max_ for reaching high transfer efficiency, we perform numerical analysis of the relation between *t*_max_ and *d*. For a given tolerable transfer error 1 − *F* = 0.5%, we plot in Fig. 6 the minimum time varies as a function of transfer distance *d*. Clearly, the time required for near-perfect transfer depends on *d* in an exponential fashion. Intuitively, this behavior results from the fact that the ground state of medium Hamiltonian 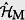 is exponentially localized due to the existence of defect *μ*_0_, leading to the exponential decrease of the energy splitting *δ*_eff_ as *d* increases; and this effect is stronger for larger *μ*_0_ and weaker for smaller *μ*_0_. In these sense, the negative effects of *t*_max_ on the transfer distance can be partially compensated by reducing the defect energy *μ*_0_. It is conceivable that *t*_max_ will scale linearly with *d* in the limit *μ*_0_ → 0.

### Robustness of state transfer

We have shown that under appropriate system parameters, the total Hamiltonian 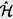 can be mapped to a three-level effective Hamiltonian 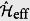, which establishes an effective STIRAP pathway for realizing long-range QST. Both efficiency and robustness are important for an information transfer scheme to be able to against technical and fundamental noises. There are two central concerns for the QST protocol in practice: decoherence and imperfect experimental implementations.

In this section, we consider a realistic model and analyze its robustness against unavoidable practical imperfections. In particular, we first consider that the Hamiltonian has a random but constant offset fluctuation in the couplings, i.e., replacing the couplings in Eq. (1b) with *J* → *J*(1 + *δε*_*j*_). The total Hamiltonian is therefore


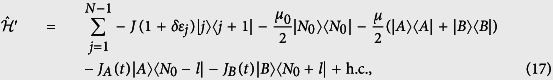


where *δ* is the maximum coupling offset bias relative to *J*; *ε*_*j*_ is drawn from the standard uniform distribution in the interval [−1, 1] and all *ε*_*j*_ are completely uncorrelated with all sites along the medium chain.

We also consider another practical situation when the Quantum Dots are subjected to phase damping due to environmental noises. As a result, the effective Hamiltonian (5) no longer holds. We now combine the two practical effects together with the modified master equation^19^


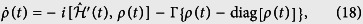


where Γ is the pure dephasing rate. To determine the robustness of the perturbed situation, we numerically integrate above equation with the electron initialized in QD-*A* to be transported: 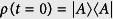. At the end of the computation (*t* = *t*_max_), we obtain the density matrix *ρ*(*t*_max_). The problem in the following we concern will be to evaluate the information transfer fidelity


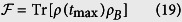


where 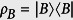.

**Figure 7:**
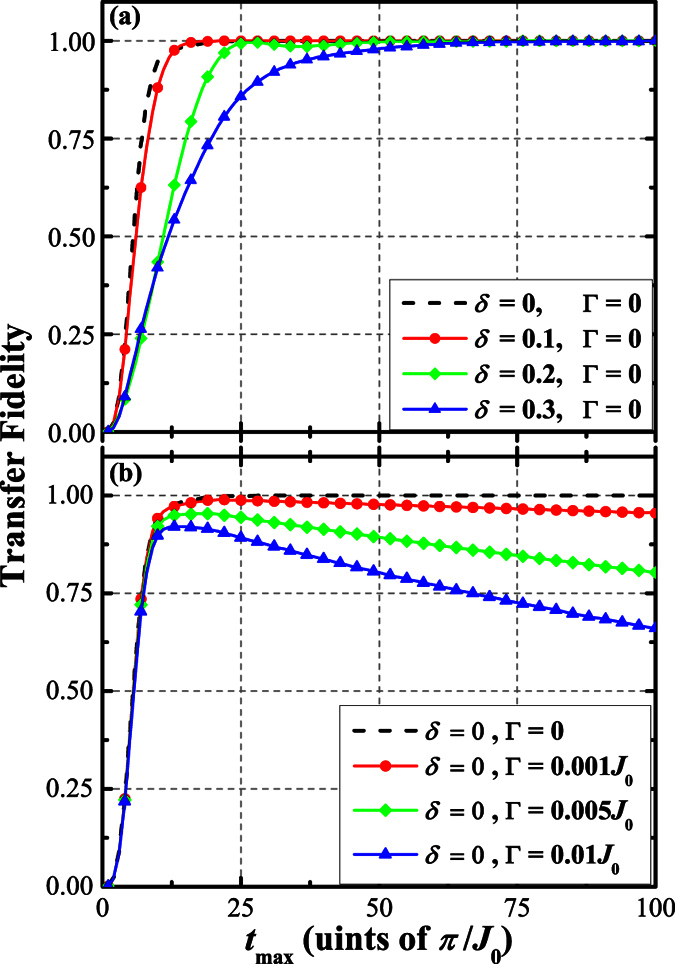
Transfer fidelity 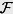 as a function of total transfer time *t*_max_ for (**a**) Γ = 0 and different values of *δ*; (**b**) *δ* = 0 and different values of Γ. The parameter values we take is *N* = 39, *μ*_0_ = 1.0*J*, *J*_0_ = 0.1*J*, and *d* = 5.

Setting *N* = 39, *μ*_0_ = 1.0*J*, *J*_0_ = 0.1*J*, and *d* = 5, Fig. 7(a) shows the solutions of the master equation (18) for Γ = 0 and for different values of maximum coupling offset *δ*, in which we have chosen to report the transfer fidelity 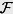 as a function of the total duration time *t*_max_ of QST. Bearing in mind that a total duration time *t*_max_ that is greater than 19*π*/*J*_0_ guarantees the perfect state transfer for ideal case (i.e., Γ = 0 and *δ* = 0). As expected, this approach is robustly insensitive to weak fluctuations (*δ* ≤ 0.1) of the couplings. By increasing *δ*, the negative effects on transfer fidelity become more and more pronounced, and this negative influence can be compensated when the duration of the process is chosen to be long enough. This means that the scheme allows one to increase the transfer fidelity arbitrarily close to unity, without the need for a precise control of the couplings.

In Fig. 7(b), we show the effects of dephasing on transfer fidelity. When dephasing is considered, perfect QST cannot be achieved. For optimum value of *t*_max_, transfer fidelity has a maximum value 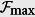 which decreases when Γ is increased. For Γ = 0.001*J*_0_, the optimum value for *t*_max_ is *t*_max_ = 21*π*/*J*_0_ with 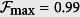. When Γ = 0.005*J*_0_, 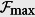 reaches 0.95 at *t*_max_ = 18*π*/*J*_0_. The optimum value of *t*_max_ is slightly shorter than that of the ideal case because dephasing will have more time to destroy the coherent transfer as *t*_max_ increases.

## Discussion

We have prosed for the first time a solid-state adiabatic quantum communication protocol that is suitable for high fidelity robust QST and quantum information swapping from one location to another. It has been shown that high fidelity two-way QST can be realized at various different distances by introducing an *N*-site tight-binding QD array as quantum data bus. We first demonstrate that the tight-binding chain with a diagonal defect has a non-vanishing energy gap above the ground state in the single-particle subspace; and this defect produces an eigenstate exponentially localized at the defect. Our approach to realize high-fidelity QST is based on the fact that the two information exchange QDs are resonantly coupled to the zeroth eigen-mode of the quantum data bus. By treating the weak coupling as perturbation, the system can be reduced to a three-level system by the first-order terms in the perturbative expansion, which enables us to perform an effective three-level STIRAP. Then we present that it is possible to transfer an arbitrary quantum state driven by adiabatically modulating two side couplings. For proper choices of the system parameters, perfect adiabatic QST can be obtained, which has been confirmed by exact numerical simulations. Moreover, for an increasing transfer distance, we find that the evolution time displays an exponential dependence on the transfer distance. However, this negative effect can be suppressed by reducing the defect energy *μ*_0_. Finally, the robustness of the scheme to fabrication disorder and dephasing is numerically demonstrated.

Comparing to the existing long-rang QST schemes, our proposal has the following advantages: i) the requirement of tunnelling control are minimized. This means that our scheme provides an efficient alternative long-range QST scheme without performing many tunnelling operations. ii) different transfer distance can be achieved by changing the connecting site of two side QDs, and no additional QDs are needed. Our proposal provides a novel scheme to implement ordinary STIRAP protocols in many-body solid-state systems in the realization of high-fidelity multiple-range QST.

## Methods

### Bethe ansatz solution of medium Hamiltonian

We will present a detail analysis of the peculiar properties of Hamiltonian 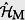 which used as a quantum channel for quantum state transfer via Bethe ansatz method^47^. The Hamiltonian is


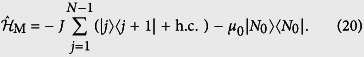


Note that the Hamiltonian 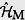 is equivalent to a tight-binding problem with single diagonal impurity at site *N*_0_. For *μ*_0_ = 0, the eigenstates are 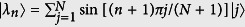 with energies *λ*_*n*_ = 2*J*cos[(*n* + 1)*π*/(*N* + 1)], and where *n* = 0, 1, 2, ..., *N* − 1. For non-zero *μ*_0_, we write the state in the single-particle Hilbert space as


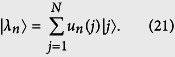


Substituting Eq. (21) into Schrödinger equation 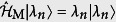, we obtain


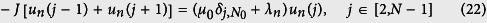


At the boundaries, we get slightly different equations:


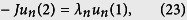



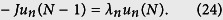


To solve above equations, we assume a usual solution by taking the mirror symmetry into consideration


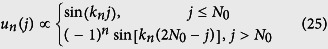


where *k*_*n*_ is the wave vector. By inserting this expression into equations (22) and together with boundary condition, we obtain the eigenvalues 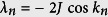 in terms of wave vector *k*_*n*_, which obeys


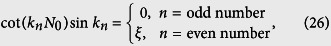


where *ξ* = *μ*_0_/2*J*.

Based on the equation (26), one can get *N* − 1 discrete real values *k*_*n*_ in the interval (0, *π*) and *one* purely imaginary wave vector. Together with the expression *λ*_*n*_ = −2*J* cos*k*_*n*_, the eigenenergies corresponding to real wave vectors are included in the band (−2*J*, 2*J*). On the other hand, the purely imaginary wave vector give rise to a out-of-band eigenenergy.

Setting *k*_0_ = *iq* and substituting it into Eq. (26), in large *N*_0_ limit we have cot*iqN*_0_ = 1 which leads 
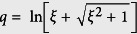
. The wave vector *q* yields the eigenvalue


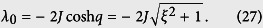


which splits off from the band. The corresponding localized state is given by 
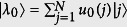
, with


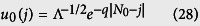


where Λ = cosh*q*/sinh*q*.

In the thermodynamic limit *N*_0_ → ∞, the excited energies become a continuous energy band; it is not hard to find that the energy gap between the ground state and the first excited state is


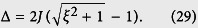


### Derivation of Effective Hamiltonian

We now reduce the full Hamiltonian (1) to an effective three-level system in the limit *J*_0_ ≪ Δ. In the absence of the coupling between the QDs A, B and the QD array (*J*_*A*_ = *J*_*B*_ = 0) the ground states of the total Hamiltonian 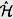 are threefold degenerate for one electron problem by setting 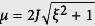, i.e., the states 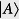, 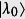, and 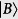 have the same energy −*μ*. According to Eqs (2) and (3), The time-dependent tunnelling rates *J*_*A*_ and *J*_*B*_ are varied in the interval [0, *J*_0_] as time process from 0 to *t*_max_. We assume the couplings between two side QDs and the bus are weak, i.e. *J*_0_ ≪ Δ, the Hamiltonian 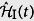 could be treated as perturbation within the time interval [0, *t*_max_]. Hence, the effective Hamiltonian can be derived by using perturbation theory at any time during the process, which acts on the subspace 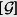 spanned by vectors 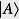, 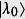, and 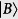.

In first-order degenerate perturbation theory, the matrix of the effective Hamiltonian with states ordering 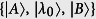 reads


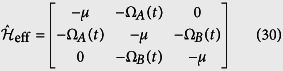


where Ω_*α*_(*t*) = *J*_*α*_(*t*)*u*_0_(*N*_0_ − *l*), for *α* = *A*, *B*. The eigenstates of the Hamiltonian of the Eq. (30) are


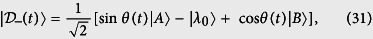



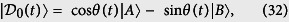



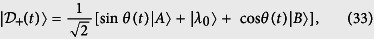


where we have introduced the mixing angle *θ*(*t*) = arctan[*J*_*A*_(*t*)/*J*_*B*_(*t*)], and corresponding energies are 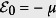, and 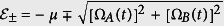.

## Additional Information

**How to cite this article**: Chen, B. *et al.* Robust Multiple-Range Coherent Quantum State Transfer. *Sci. Rep.* **6**, 28886; doi: 10.1038/srep28886 (2016).
